# The Affective Norms for Polish Short Texts (ANPST) Database Properties and Impact of Participants’ Population and Sex on Affective Ratings

**DOI:** 10.3389/fpsyg.2017.00855

**Published:** 2017-05-30

**Authors:** Kamil K. Imbir

**Affiliations:** Faculty of Psychology, University of WarsawWarsaw, Poland

**Keywords:** short texts, affective norms, valence, arousal, dominance, origin, subjective significance, source

## Abstract

The Affective Norms for Polish Short Texts (ANPST) dataset (Imbir, 2016d) is a list of 718 affective sentence stimuli with known affective properties with respect to subjectively perceived valence, arousal, dominance, origin, subjective significance, and source. This article examines the reliability of the ANPST and the impact of population type and sex on affective ratings. The ANPST dataset was introduced to provide a recognized method of eliciting affective states with linguistic stimuli more complex than single words and that included contextual information and thus are less ambiguous in interpretation than single word. Analysis of the properties of the ANPST dataset showed that norms collected are reliable in terms of split-half estimation and that the distributions of ratings are similar to those obtained in other affective norms studies. The pattern of correlations was the same as that found in analysis of an affective norms dataset for words based on the same six variables. Female psychology students’ valence ratings were also more polarized than those of their female student peers studying other subjects, but arousal ratings were only higher for negative words. Differences also appeared for all other measured dimensions. Women’s valence ratings were found to be more polarized and arousal ratings were higher than those made by men, and differences were also present for dominance, origin, and subjective significance. The ANPST is the first Polish language list of sentence stimuli and could easily be adapted for other languages and cultures.

## Introduction

Sets of normative affective stimuli are a popular and useful research tool. They provide stimuli with reliably measured, known affective properties. Such stimuli include images [e.g., pictures from the International Affective Picture System (IAPS; Lang et al., 2008)], sounds [e.g., from the International Affective Digital Sounds (IADS; Bradley and Lang, 1999b)], verbal or symbolic representations of objects [e.g., words from the Affective Norms for English Words (ANEW; Bradley and Lang, 1999a), and sentences (e.g., from the Affective Norms for English Text (Bradley and Lang, 2007)]. The growing interest in verbal research materials resulted in adaptations of norms for words for languages other than English, such as European Portuguese (Soares et al., 2012), Spanish (Redondo et al., 2007; Ferré et al., 2012; Hinojosa et al., 2016), French (Gilet et al., 2012; Monnier and Syssau, 2013), Italian (Montefinese et al., 2013, 2014), German (Võ et al., 2006, 2009; Grühn and Smith, 2008; Lahl et al., 2009; Kanske and Kotz, 2010; Schmidtke et al., 2014; Citron et al., 2015), Finnish (Eilola and Havelka, 2010), Dutch (Moors et al., 2013), and Polish (Imbir, 2015, 2016a; Riegel et al., 2015; Wierzba et al., 2015).

Interest in the processing of affectively charged stimuli has been growing since Osgood et al. (1957) research on semantic differentials. Their idea was simple, namely to put stimuli (e.g., words) into assessments of irrelevant qualities based on many bimodal dimensions (such as soft vs. taught). These data were subjected to factor analysis, which revealed that the variance in ratings could be attributed to three main factors, namely valence, arousal, and power/dominance. Based on Osgood et al. (1957) findings, Lang (1980) developed the Self-Assessment Manikin (SAM), allowing someone to rate the affective quality of stimuli without using language. The SAM is a schematic human figure that can be used to represent symbolically the states associated with different points on a Likert scale. It is especially important to minimize the verbal requirements of scales used to assess affective reactions to stimuli, since the verbalization of affect change its quality (Frijda, 2007).

Valence is determined by whether a stimulus elicits a positive or negative reaction (Moors et al., 2013) and determines the polarity of emotional activation (Lang et al., 1997). This dimension is the easiest one to assess; in affective norms studies assessments of valence are usually characterized by the best reliability and stability of measurement estimations (e.g., Soares et al., 2012; Monnier and Syssau, 2013; Moors et al., 2013; Imbir, 2015, 2016a). Arousal is a measure of energy level or intensity of feelings (Montefinese et al., 2014) and also captures excitement or bodily activation (Lang et al., 1997). Arousal enables an organism to cope with dangerous situations (cf. fight or flight reaction) or engage in appealing interactions with potential sexual partners. It has also been hypothesized that arousal modulates the balance between heuristic and systematic processing, shifting it toward simpler experiential mind mechanisms (Epstein, 2003). The dominance variable is sometimes referred to as control (Montefinese et al., 2014) or power (Moors et al., 2013) and captures the subjective impression of how controllable affective reaction to the stimulus is. Measurements of this dimension are usually less reliable than measurements of valence or arousal (Imbir, 2015), they explain less variance (Lang et al., 1997) and so dominance is used less frequently when sets of affective norms are developed (e.g., Gilet et al., 2012; Monnier and Syssau, 2013; Riegel et al., 2015).

The valence and arousal factors appeared to shape individuals’ reactions to emotional stimuli. For example, valenced words, both negative and positive, were found to elicit faster lexical decisions than neutral words (Kousta et al., 2009; Yap and Seow, 2014; Kuperman, 2015). Arousal was found to modulate processing of words: highly arousing stimuli have been shown to evoke a larger amplitude of event-related potentials in comparison to less arousing stimuli (e.g., Cuthbert et al., 2000; Dillon et al., 2006; Fischler and Bradley, 2006; Gibbons, 2009). Arousal was also shown to influence reaction times in lexical decision tasks (faster responses for highly arousing stimuli), incidental memory (better recollection for highly arousing words) and pupillary response (highly arousing words elicit a smaller pupillary response: Bayer et al., 2011). Other studies have reported that valence and arousal interact during visual word recognition and lexical decision-making (Citron et al., 2014a,b; Recio et al., 2014; Vinson et al., 2014).

### The Emotion–Duality Model and its Consequences

The nature of emotions is one of the biggest puzzles for psychology in the 21st century. At present, there is no consensus about what an emotion is (Kagan, 2007). In some of the cases, dimensional approaches, exemplified by the use of affective norms, provide an answer to this difficulty. The assumption is simple: rather than focusing on discrete affective states we can investigate underlying factors such as valence, arousal, and dominance; however, this does not resolve all the questions about the diversity of emotions. Some psychologists insist on a categorical approach, arguing that discrete emotions such as happiness, anger, sadness, fear, or disgust (cf. Stevenson et al., 2007; Wierzba et al., 2015) contribute significantly to affective word processing.

Another perspective on how the mind works is the duality of mind approach (for review see: Gawronski and Creighton, 2013), which compares uncontrolled and controlled mental processes involved in behavior production. This approach has been applied in different domains, including cognition (e.g., Evans, 2003; Kahneman, 2003, 2011; Lieberman, 2003; Deutsch and Strack, 2006), social cognition (e.g., Bargh, 1994; Moors and De Houwer, 2006) and research on personality structure (e.g., Epstein, 2003). Recently, the duality of mind approach has been adopted by researchers investigating emotional processes (Jarymowicz and Imbir, 2015), who distinguished between the automatic emotional system (AES) and the reflective emotional system (RES). Both systems cover a huge range of emotions and each may be triggered by two main sources of emotional stimulation, internal, and external.

In the AES, emotions originate in non-verbalized, immediate biological processes of appraising internal state (e.g., homeostasis monitoring) and the external world (incentives and disincentives or punishments). These automatic appraisals are based on biological criteria of evaluation (Damasio, 2010) and provides some fundamental mechanisms of regulation giving a sense of what is bad or good from an evolutionary fitness point of view. In the AES, anything which increases biological fitness (e.g., relaxation, calorific food, calm, sunny weather, release) is evaluated positively, whereas events that threaten biological fitness (e.g., danger, famine, stress, stormy weather, predator) are evaluated negatively. The AES learns slowly, hence the immediate pleasure of drugs can override the long-term suffering that will result from addiction; it is hard to achieve a long-term perspective using the AES.

In the RES, emotions originate in a verbalized evaluation criterion of conscious appraisal of the reality with use of evaluative standards (Reykowski, 1989). They are based on norms of what is good or bad and are developed on the basis of (1) AES activity (verbalization of bodily reactions), (2) social influences, and (3) pure cognitive reasoning processes. Norms may concern internal standards of behavior or performance but they also extend to the external word-engaging axiological sense of what is good or bad (e.g., standards concerning positive evaluations of justice, including even volunteering to pay our parking fines). The RES relies on human cognitive processes such as deliberate thinking, abstraction, conceptual thought, or representation of past and future states to create a temporal perspective (Imbir, 2013). Together, these processes mean that the evaluation process is highly plastic, depending, for example, on which standard is active at the moment of evaluation. The RES allows a person to modify and to adapt AES reactions in accordance with aims and expectations about the future. Jogging regularly (perceived as tiring or boring by the AES) may be treated as a pleasurable activity, as something that helps one maintain one’s fitness for decades.

Two new dimensions based on the emotion duality model have been proposed. The first is origin of an emotional state, from “heart” vs. “mind.” This dimension is based on the assumption that some emotions elicited by a stimulus emerge immediately and without explicit thought whereas other are the result of careful consideration. From a theoretical point of view the two origin systems use qualitatively different processes (cf. Epstein, 2003; Evans, 2003; Kahneman, 2003, 2011; Lieberman, 2003; Deutsch and Strack, 2006; Gawronski and Creighton, 2013; Jarymowicz and Imbir, 2015; Imbir, 2016b); however, in research on affective norms (Imbir, 2015), origin has been operationalized as a continuum in a similar way to valence. The rationale for this is that participants using a SAM scale do not assess the systems actually engaged in processing (which are not accessible to subjective experience); instead they rate the perceived ratio of engagement of the two systems. There are certainly some states that are easily associated with automatic or controlled processing but there are many more mixed states that are hard to label subjectively. The Origin scale is intended to measure subjective perceptions of the relative engagement of the AES and RES, as it is assumed that this is the best currently available method of tapping the mechanisms underlying both systems.

The second new dimension is source of emotional state (internal vs. external) operationalized as subjectively perceived locus of causes (Jarymowicz and Imbir, 2015). In both the AES and RES, the causes of emotional states can be located inside or outside the body. This does not imply that an emotional state can be located outside the body, simply that the event or stimulus responsible for it can be internal or external. As in the case of origin, source is theoretically a dichotomous rather than continuous variable; however, the source of emotions may be perceived as indeterminate or a mixture of internal and external loci and so in practice it is easier to assess it using a single scale; this also means that ratings of source can be compared with ratings of valence. Both new scales were shown to be sufficiently reliable (Imbir, 2015, 2016a) that their use in another affective norm study seemed warranted.

Some preliminary data suggest that factors such as the origin and source of emotions modulate the scope of attention (Imbir, 2013), widening it in the case of reflective or external stimuli. Participants were required to detect and react to a stimulus (small red dot) presented on a screen at a variable distance from a central fixation point. After degraded (very brief, about 32 ms, and then masked) presentation of words or reading at own pace sentences (and imagining that situation described in the sentence happened to the participants) associated with reflective origin or external source, subjects reacted to stimuli distant from the fixation point as fast as to stimuli close to fixation points. In the case of automatic origin or internal source elicitation, detection times for the stimuli distant from the fixation point were longer than those for close stimuli. Other data suggest that origin influences cognitive control (Imbir and Jarymowicz, 2013), making it difficult to maintain control after presentation of stimuli that elicit automatic affective responses. This research was carried out using the Emotional Stroop Test (for word stimuli) or antisaccade task (for sentence stimuli). In both cases reaction times were longer for stimuli which elicited an automatic affective reaction than those which elicited a reflective reaction. Recently, origin was found to modulate the late positive component of event-related potential correlates of emotional word processing, regardless of whether meaning was processed explicitly (Imbir et al., 2015) or implicitly (Imbir et al., 2016). In the explicit processing condition, participants were required to read words and decide whether they were emotional or neutral. During the 420–780 ms window, after stimulus presentation we observed that one independent component in the left-parietal region had a higher amplitude (Imbir et al., 2015) after affectively valenced automatic words than after reflective words, although it was not different from that elicited by neutral words. A lexical decision task was used in the experiment involving implicit processing (Imbir et al., 2016). Differences caused by origin of an affective state associated with connotations of verbal stimuli were found in the Late Positive Complex component of Event Related Potential. The pattern of differences and scalp localization were similar to those found for explicit word processing (Imbir et al., 2015). These results suggest that the duality of emotion approach may shed new light on the cognitive consequences of emotional processing on cognition, and so it is important to establish new methods of manipulating the origin and source of affective reactions to stimuli precisely.

### System-Specific Mechanisms of Activation

The duality of emotion approach (Jarymowicz and Imbir, 2015) assumes that there are different mechanisms underlying activation of the AES and RES (Imbir, 2016b). This assumption is based on the claim that activation selectively improves processing characteristics for each mental system (AES or RES), but decreases processing characteristic for the other mental system (Epstein, 2003). Arousal is postulated to be an activating mechanism specific to non-verbal AES processing, because arousal improves only simple mental processes (such as detection of danger or the immediate response to danger). In experiential vs. rational mind theory (Epstein, 2003), it is suggested that increasing arousal level is the main factor capable of shifting the balance between both minds toward the experiential system. Although Epstein’s model applies to personality and cognition rather than emotion, we would argue that AES processing is similar to the mechanisms of the experiential mind. When we see something tasty we become more aroused and this motivates us to approach the tasty, delicious object. No thinking is required in such circumstances.

But what might activate RES processing? Such processing requires a lot of resources because it is based on propositional processes (cf. Kahneman, 2003; Strack and Deutsch, 2004; Deutsch and Strack, 2006; Imbir, 2016b). The question is, in what circumstances do people invest resources in slow processing (Kahneman, 2011)? We believe that subjective significance is the analog of arousal for the RES. When an individual detects something with important implications for his or her goals, expectations and needs – i.e., something with subjective significance – it motivates the individual to act. Arousal increases as soon as we are confronted with a biologically relevant situation, whereas subjective significance is attributed consciously, and thus that attribution occurs more slowly. There are several results relating to the willpower and ego depletion effect (e.g., Baumeister et al., 1998), which show that being willing to do something improves performance, but this costs a lot of energy, hence performance on tasks requiring self-control is higher after drinking soda containing normal sugar than after drinking sugar-free soda with few calories. Recently a reliable scale for measuring subjective significance was developed (Imbir, 2015). This scale has a similar format to scales measuring the arousing properties of stimuli; it assesses the extent to which stimuli are associated with personally meaningful or important experiences. When used to derive word norms (Affective Norms for Polish Words: ANPW: Imbir, 2015), the arousal and subjective significance scales showed similar reliability. Stimuli from the ANPW were used to test the role of dual activation mechanisms: subjective significance and arousal, for interference control in the Modified Emotional Stroop paradigm (Imbir, 2016c). It appeared that subjective significance reduced the increase in reaction latencies caused by arousal.

### Reasons for Creating the ANPST and Assessing Group as well as Sex Differences

The motivation for creating the Affective Norms for Polish Short Texts (ANPST) was to provide short texts with known, reliable affective ratings with respect to standard affective dimensions (valence, arousal, and dominance) and new affective dimensions related to the emotion–duality model (Jarymowicz and Imbir, 2015), namely origin, subjective significance, and source. Sentences are a useful method of eliciting complex affective processing because they contain contextual information involved in the sentence structure. Until now there were only a few databases containing such research materials (e.g., Affective Norms for English Texts: Bradley and Lang, 2007). The Affective Norms for English Texts dataset provides normative ratings of perceived valence, arousal and dominance (measured with use of SAM scales) for a set of sentences and brief texts in the English language. Recently the Minho Affective Sentences dataset (Pinheiro et al., 2016) was introduced; this is a set of 192 sentences covering five basic emotional states (anger, fear, disgust, sadness, and happiness) and control/neutral stimuli in European Portuguese. Sentences were assessed by 536 native speakers of European Portuguese with respect to valence, arousal, and dominance using nine-point SAM scales (Pinheiro et al., 2016). Minho Affective Sentences assessments were found to replicate the quadratic relationship between valence and arousal in affective space.

The aim of the ANPST study was to check the affective meaning of the short text materials. Additionally the type of student sample as well as the impact of sex on affective reactions to ANPST words were subjects of specific interest. For that reason, two samples were investigated: (1) female psychology students, as they often participate in psychology experiments and (2) a general student sample drawn from other departments and various colleges. The differences between psychology students and other students were expected, based on the assumption that a particular personality profile associated with choosing psychology as a future career is present: careers in psychology demand empathy, understanding, sensitivity to emotions, etc., and hence one would expect psychology students to be more receptive to affectively charged materials. There is no published evidence for this hypothesis (to the author’s knowledge there are no studies explicitly comparing psychology students and other students), but it is quite commonly held to be valid by researchers teaching in psychology departments. An exploratory approach was applied toward this issue, thus no specific expectations were formulated toward differences between groups.

In the non-psychology student population a comparable number of females and males were invited into the study. The aim for this was to check whether there were differences in the way emotional stimuli were perceived by women and men. In normative studies for affective stimulus fields, there is still a debate concerning the role of sex in affective reactions to emotional stimuli. Some studies shows that sex differences exist in affective reactions to stimuli (Monnier and Syssau, 2013; Montefinese et al., 2014), but other studies report no such differences (Redondo et al., 2007). Acknowledging inconsistency in results of previous studies, no specific expectations were formulated toward sex differences.

Finally, the aim of the ANPST was to assess relationships between measures found in an earlier study based on word stimuli (Imbir, 2015). This is especially important, because evaluation of the emotion duality model may be provided in the context of methodology used for affective norms collection. These norms allow one to measure the structure of affect by means of measuring relations between different affective dimensions. Results collected in the case of the ANPST were expected to show similar correlations between the six affective dimensions to those obtained in previous work on collection of word norms (Imbir, 2015, 2016a).

## Materials and Methods

### Participants

Final ratings reported in the ANPST (Imbir, 2016d) were based on 2,239 completed questionnaires. Participation was voluntary and unpaid. Researchers instructed the participants that their assessments would make an important contribution to future research. Assessments in Studies 1a and 1b were performed for a subset of 108 sentences. In Study 1a, they were performed by 148 psychology students (women only, aged from 19 to 39 years: *M* = 21; *SD* = 2.08) and in Study 1b by 322 students (179 women, 143 men) enrolled in various departments (social science, engineering, life science, and science) of Warsaw colleges and universities (aged from 18 to 40 years: *M* = 22; *SD* = 2.28). Assessments in Study 2 were performed for a subset of 610 sentences by 1,769 students (882 women and 887 men) from various departments (social science including psychology, engineering, life science, and science) of Warsaw colleges and universities (aged from 18 to 47 years: *M* = 20; *SD* = 1.88).

### Materials and Procedures

The full set of 718 emotive sentences in Polish was assessed in Studies 1 and 2 (Imbir, 2016d). The sentences were either taken from previous research (*n* = 108; Imbir, 2013; Imbir and Jarymowicz, 2013) or collected for the ANPST directly from various sources (*n* = 610 additional sentences; Imbir, 2016d). Sentences were modified in a rather non-specific manner (Frijda, 2007) to enable the participants to relate the content to their lives and experiences (see dataset). **Table 1** presents examples of sentences from a dataset that scored the most extreme on assessments.

**Table 1:** Examples of sentences from the dataset with the most extreme ratings for each of evaluated dimension.

English version	Polish version	Valence *M*	Arousal *M*	Dominance *M*	Origin *M*	Subjective significance *M*	Source *M*
Saving a person’s health or life after a complicated operation is a great joy!	Uratowanie zdrowia czy życia po skomplikowanej operacji to wielka radość!	8.16					
It is pleasurable to kiss a loved one with abandon.	Przyjemnie jest całować ukochaną osobęe bez opamięetania.	8.13					
In near-death moments, a person is struck by an awareness of a hollowness that cannot be filled.	W momencie śmierci bliskiego uderza człowieka świadomość niczym nie dającej sięe zapełnić pustki.	1.65					
The old lady was badly beaten because she commented on the boy’s behavior at the bus stop.	Staruszka została dotkliwie pobita, ponieważ zwróciła chłopakowi uwagęe na przystanku.	1.67					
The mother looked at her child with disgust, then began to beat it violently.	Matka z obrzydzeniem spojrzała na swoje dziecko, po czym zaczęeła je gwałtownie bić.		7.77				
The aroused pedophile watched the unsuspecting children, playing unsupervised on the neighborhood playground.	Podniecony pedofil obserwował nieświadome niczego dzieci bez opieki bawiące sięe na osiedlowym placu zabaw.		7.77				
In the winter, brown bears fall into a state of inactivity called hibernation.	Zimą niedźwiedzie brunatne zapadają w stan spoczynku nazywany hibernacją.		1.86				
Water freezes at 0 degrees Celsius.	Woda zamarza w temperaturze 0 stopni Celsjusza.		2.12				
Saving a person’s health or life after a complicated operation is a great joy!	Uratowanie zdrowia czy życia po skomplikowanej operacji to wielka radość!			7.93			
Make your weakness your strength, and it will stop being your weakness.	Uczyń ze słabości swoją siłęe, a wtedy przestanie to być twoim słabym punktem.			7.48			
Losing someone close provokes not only mental but also physical suffering.	Strata kogoś bliskiego wywołuje nie tylko psychiczne, ale i fizyczne cierpienie.			2.24			
John had such a high fever that he got a rash and began to vomit.	Jan miał tak wysoką gorączkęe, że dostał wysypki i zaczął wymiotować.			2.19			
Wood undergoes a combustion reaction at high temperatures.	Drewno w wysokiej temperaturze ulega reakcji spalania.				8.2		
A line can be divided into equal parts with a compass.	Podział odcinka na równe częeści jest możliwy przy użyciu cyrkla.				8.17		
She loved her husband so much she gave her life for him.	Tak bardzo kochała męeża, że oddała za niego swoje życie.				1.85		
From between the lips, from the corners of the mouth, streamed the sweetness of swaying sensuality, the hot lust of pleasure.	Mięedzy wargami, przez kąciki ust, płynęeła, sama słodycz rozkołysanej zmysłowości, gorąca żądza rozkoszy.				1.83		
After the accident, I was diagnosed with a spinal fracture, both broken tibia, and a skull fracture.	Po wypadku zdiagnozowano u mnie złamanie kręegosłupa, obu goleni oraz kości czaszki.					7.98	
John was aggressive to the point that he beat his own daughter to death.	Jan był agresywny do tego stopnia, że pobił śmiertelnie własną córkęe.					7.71	
The lawn on the right side of the path looked unmown, but there was no sign of dandelions, daisies, or moss.	Trawnik po prawej stronie ścieżki wyglądał na nieskoszony, ale nie było śladu mleczy, stokrotek czy mchu.					2.15	
Alicia had a black and white cat named Philemon.	Alicja ma biało czarnego kota o imieniu Filemon.					1.87	
The formation of glaciers is possible in the high mountains.	W wysokich górach możliwe jest tworzenie sięe lodowców.						7.4
Forests produce oxygen, cleanse the air, and muffle noise.	Lasy produkują tlen, oczyszczają powietrze oraz tłumią hałas.						7.32
Hunger is real when someone looks at other people as something to eat.	Głód jest wtedy prawdziwy, gdy człowiek patrzy na drugiego człowieka jako na obiekt do zjedzenia.						2.51
One can be disappointed in one’s own behavior.	Własnym zachowaniem można doprowadzić do poczucia rozczarowania sobą.						2.42

All the sentences from the dataset were translated into English by a bilingual philologist who has spent considerable time working and living in the United States and thus has a deep knowledge of American culture and customs. The English sentences were then back-translated by another bilingual person who specializes in the English language. Finally a third person, a native Polish-speaking competent judge rated the congruence in meaning of the original and back-translated sentences. In 695 cases this judge approved the translations as maintaining the original meaning, the English translations of the remaining 23 cases (3.2%) were amended to achieve satisfactory congruency. Using this procedure ensured the English versions of the original Polish sentences shared the same meaning. The original Polish sentences varied in length from 5 to 23 words (*M* = 11.69; *SD* = 3.04) and from 36 to 133 letters (*M* = 77.48; *SD* = 18.57). Values for the frequency of appearance in Polish of the words used in the sentences were taken from subtlex-pl (Mandera et al., 2015), a huge corpus of Polish words giving the number of times they appear in a large database of movie and television program subtitles. These values were used to calculate mean and median frequency separately for all sentences. The mean natural logarithm (LN) of word frequency was also calculated, as the raw frequency data were very skewed. The LN-transformed mean frequency for the original Polish sentences varied from 4.98 to 13.36 (*M* = 9.38; *SD* = 1.25).

**FIGURE 1:**
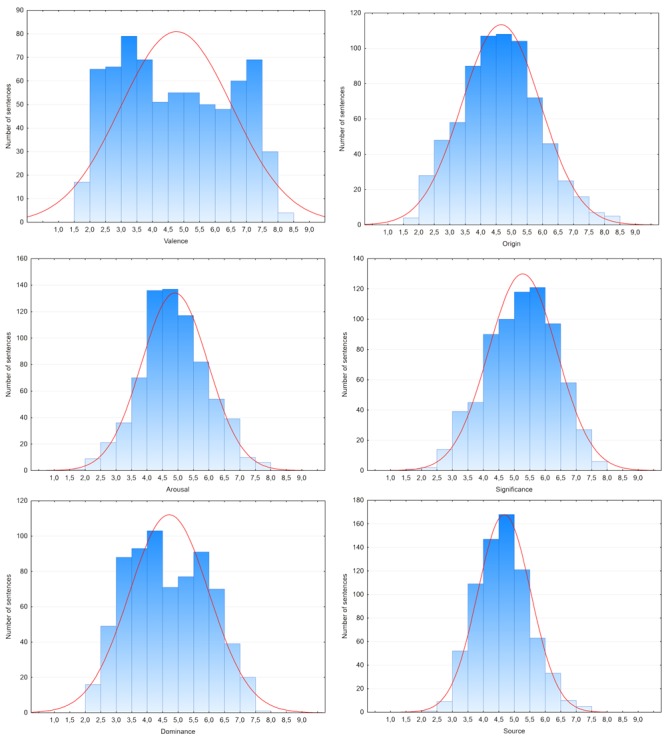
**Frequency histograms of ratings for all dimensions**.

Six SAM scales were used to assess the affective meaning of the sentences, three (valence, arousal, and dominance) were adapted from Lang (1980) and the remaining three (origin, significance, and source) were created specifically for research on the emotion–duality model (Imbir, 2016b). The reliability of the scales was assessed as part of a study collecting ANPW (Imbir, 2015, 2016b). The results indicated that the reliability of the valence, arousal, dominance, origin, and subjective significance scales was satisfactory (Pearson correlations between different samples ranged from 0.73 to 0.99); the reliability of the source scale was worse, although still adequate (correlations ranged from 0.62 to 0.77). All SAM scales consisted of five schematic representations of people displaying different degrees of emotion, ranging from one pole of a dimension to the other (c.f. Figure 1 in Imbir, 2016d).

The procedure was the same for Studies 1a, 1b, and 2 (c.f. Imbir, 2016d). The studies differed only in the nature of the sample (1a vs. 1b and 2) and the exact materials used (1a and 1b vs. 2). Sentence assessments were carried out using a paper-and-pencil procedure. Groups of participants assessed the sentences in sessions which took place in seminar or lecture rooms. In each session, the researchers explained the aim of the study to the participants before they started assessing the sentences. The entire procedure took no more than 25 min. No time limit was set, but participants were encouraged to answer as quickly as possible, as we were interested in their first impression of the affective state each sentence evoked. Participants’ confidentiality was assured, but after they had completed the assessments they were asked to complete a socio-demographic questionnaire asking about sex, age, department, and number of years of university or college education. The details concerning procedures applied can be found in the ANPW dataset manuscript (Imbir, 2016d).

## Results and Discussion

### ANPST Descriptive Statistics and Reliability Estimations in the Context of Norms for Words

Descriptive statistics (number of assessments, *N*; Mean, *M*; Standard Deviation, *SD*; Range) for all the sentences were calculated separately for each of the six scales in Studies 1a, 1b, and 2. Descriptive statistics for all of the participants’ assessments in both studies were also calculated. All normative values for valence, arousal, dominance, origin, significance, and source assessments in individual studies and in all studies combined are contained in a dataset^1^. All analyses were carried out using IBM SPSS 23 statistical software. The figures were generated using STATISTICA 14.5 software. **Table 2** presents descriptive statistics for the combined assessments of all affective variables and lexical dimensions (word count, number of letters in the sentence, median LN frequency of words making up the sentence).

**Table 2:** Summary of variables included in the word list with means (M), standard deviations (SD), and ranges for all participants, non-psychology female and male students.

Affective dimension	ALL			Females (psychology)			Females (non-psychology)			Males (non-psychology)		
	*M*	*SD*	*Range*	*M*	*SD*	*Range*	*M*	*SD*	*Range*	*M*	*SD*	*Range*
Valence	4.74	1.77	1.65–8.16	5.06	2.18	1.38–8.43	4.67	1.98	1.19–8.46	4.82	1.58	1.73–8.46
Arousal	4.88	1.07	1.86–7.77	5.23	2.21	1.61–7.8	5.05	1.20	1.87–8.42	4.68	1.03	1.53–7.52
Dominance	4.69	1.28	2.19–7.93	4.9	2.08	1.7–7.92	4.61	1.47	1.56–8.32	4.76	1.16	2.19–7.62
Origin	4.64	1.26	1.83–8.24	4.57	2.25	1.52–8.7	4.59	1.44	1.46–8.33	4.70	1.16	1.81–8.45
Significance	5.25	1.1	1.87–7.98	5.89	1.95	1.17–8.41	5.37	1.27	1.85–8.15	5.08	1.02	2.16–7.92
Source	4.65	0.85	2.41–7.40	4.6	2.39	2.22–8.11	4.64	0.98	1.89–7.90	4.68	0.85	2.21–7.15
Number of words	11.69	3.04	5–23									
Number of letters	77.48	18.57	36–133									
Mean LN of frequency	9.38	1.26	4.98–13.36									

The distribution and homogeneity of the participants’ affective ratings was the first point of interest (c.f. Lahl et al., 2009; Fairfield et al., 2017). Participants assessed sentences using a nine-point Likert scale where 1 represented negative/calm/being in control/from the heart/of no consequence/internal, and 9 meant positive/excited/controlling/from the mind/important/external for the valence, arousal, dominance, origin, subjective significance, and source scales, respectively. In all SAM scales 5 was described as a neutral/mixed/moderate state. The three distributions deviated significantly from a normal distribution (Kolmogorov–Smirnov test: *D*s > 0.04, *p*s < 0.001), namely valence, dominance, and significance, while arousal origin and source distributions did not differ significantly from a normal distribution (Kolmogorov–Smirnov test: *D*s < 0.04, *p*s > 0.05). Kurtosis was -1.23 for valence, -0.14 for arousal, -0.94 for dominance, -0.22 for origin, -46 for subjective significance and -0.05 for source. Skewness was slightly positive for valence (0.14), arousal (0.11), dominance (0.15), origin (0.19), and source (0.28) and slightly negative for subjective significance (-0.19). It appeared that in the case of valence, the distribution was bimodal, while in the case of all other dimensions it was unimodal. Also, as can be seen in **Table 2** and on **Figure 1**, it is worth noting that in all of the cases, except for the source scale, the affective ratings covered almost the entire rating scale used. **Figure 1** presents frequency histograms for all dimensions (classical and new proposed dimensions).

**FIGURE 2:**
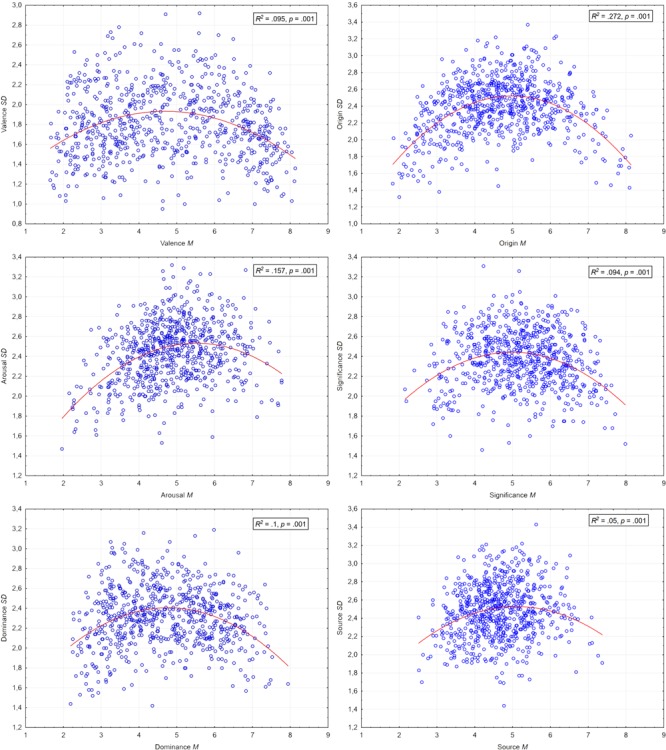
**Homogeneity of assessments: means vs. standard deviations for all dimensions**.

Considering the homogeneity of ratings, the distribution of assessments was plotted against standard deviations. Additionally, regression lines with *R^2^* and *p*-values for each case are provided. Ratings’ distribution in *M* × *SD* space gives us information concerning to what extent assessments were congruent. It is especially important for neutral/moderate (around the middle of the scale) assessments that may be the result of (a) neutral or moderate properties of the stimulus when *SD* is low or (b) incongruent assessments, when some participants rate the stimulus as low whereas other participants rate it as high in certain measures. It appears that in each case the quadratic function describes best plotted relationships. This means that among neutral/medium stimuli only some were rated in a congruent, moderate way, whereas the rest were perceived once as representing one end of the scale by some participants, twice as representing the other end of this scale by other participants. High SDs indicate incongruence in assessments of certain sentence. Considering *R*^2^ values, such a relationship is especially strong for origin and then surprisingly arousal dimensions, but much less for source (see Introduction section), significance or valence. It is important to consider *SD* values when neutral stimuli from ANPST are of interest. **Figure 2** represents the homogeneity of ratings for all dimensions; means for the sentence assessments are plotted against the standard deviation.

Reliability was assessed using the split-half procedure (Imbir, 2016d). Pearson correlations were significant in all cases (*p* < 0.001) and varied from 0.935 for valence to 0.657 for source (c.f. **Table 3**). The split-half method underestimates reliability because the original sample is split into two subsamples so the Spearman–Brown formula (see, Riegel et al., 2015) was applied to raw correlations. The inspection of **Table 3** allows us to conclude that scales used in other affective norm studies demonstrated similar reliabilities in the sentence assessments carried out for this study; once again, reliability was worst for the source scale; however, based on these values one can still conclude that in the context of the ANPST the source scale was reliable (adjusted *r* = 0.793). **Table 3** presents estimates of the reliability of the six affective scales compared to reliabilities of those scales measured for norms for words in ANPW (Imbir, 2015) and ANPW_R (Imbir, 2016a).

**Table 3:** Reliability estimates (Pearson correlations between ratings given for each variable on two versions of questionnaire) for the ANPST (Imbir, 2016d) in comparison to reliability estimates for the ANPW (Imbir, 2015) and the ANPW_R (Imbir, 2016a).

Scale	Split-half correlation in ANPSP (Imbir, 2016d): (Sperman–Brown correction)	Split-half correlation in ANPW (Imbir, 2015)	Split-half correlation in ANPW_R (Imbir, 2016a): (Sperman–Brown correction)
Valence	0.935 (0.966)	0.95	0.973 (0.986)
Arousal	0.755 (0.86)	0.78	0.841 (0.914)
Dominance	0.850 (0.919)	0.78	0.868 (0.929)
Origin	0.815 (0.899)	0.73	0.828 (0.906)
Significance	0.745 (0.855)	0.78	0.852 (0.92)
Source	0.657 (0.793)	0.62	Not assessed

The results of research on the ANPW (Imbir, 2015), ANPW_R (Imbir, 2016a) and ANPST suggests that participants are able to provide coherent ratings of the proposed new dimensions and that ratings are consistent across different populations. The consistency is most impressive in the case of origin, followed by subjective significance, and is lowest for the source dimension. This may reflect the comprehensibility and ease of use of the proposed new dimensions. Origin is described using the well-known (in Western culture) metaphorical distinction between heart and mind. Subjective significance is the personal significance of an evoked experience or state and is thus fairly easy to understand and use. The source dimension is harder to use, however, because it is hard to say if a particular affective state has its source in external events or internal stimuli because emotions are embodied and sensations are inherently internal.

### Correlations between Variables

Pairwise Pearson’s correlations were calculated to assess relationships between all variables included in the ANPST. Full results are presented in **Table 4**; here, only significant correlations with *r* > 0.2 are listed (sharing more than about 4% common variance). In the case of correlations between the classical dimensions measured in the ANPST, two relations appeared to be significant, the negative correlation between valence and arousal (*r* = -0.28, but note below), and the strong positive relation between dominance and valence (*r* = 0.84, *p* = 0.001). There were also two other considerable correlations between the new and classical dimensions: a negative one between origin and arousal (*r* = -0.36, *p* = 0.001) and a positive one between subjective significance and arousal (*r* = 0.52, *p* = 0.001). Correlations between the new dimensions were rather weak (all were lower than *r* < 0.35, thus they represented less than about 10% common variance shared by related dimensions). Origin and source were positively correlated (*r* = 0.33, *p* = 0.001) and there were negative correlations between subjective significance and origin (*r* = -0.28, *p* = 0.001) and between subjective significance and source (*r* = -0.2, *p* = 0.001). **Table 4** presents the full pattern of correlations. Non-linear relations (c.f. below) are indicated by a shaded cell background.

**Table 4:** Pearson correlations between the variables based on all participants’ ratings of 718 sentences.

	Arousal	Dominance	Origin	Significance	Source	Number of words (length)	Number of letters (length)	Mean LN of word frequency
Valence	**–0.28^∗∗^**	**0.83^∗∗^**	**–0.16^∗∗^**	–0.01	**–0.08^∗^**	–0.04	–0.02	0.01
Arousal		**–0.13^∗∗^**	**–0.36^∗∗^**	**0.51^∗∗^**	–0.03	0.07	0.09^∗^	0.03
Dominance			–0.09^∗^	0.09^∗^	**–0.11^∗∗^**	–0.04	–0.001	–0.01
Origin				**–0.28^∗∗^**	**0.33^∗∗^**	**–0.13^∗∗^**	**–0.12^∗∗^**	**–0.1^∗∗^**
Significance					**–0.20^∗∗^**	0.03	0.05	**0.15^∗∗^**
Source						–0.08^∗^	–0.04	**–0.24^∗∗^**
Number of words (length)							**0.87^∗∗^**	**0.28^∗∗^**
Number of letters (length)								–0.06

*Darkened correlations indicates that quadratic relation better explains the variance. ^∗∗^*p* < 0.001; ^∗^*p* < 0.05.*

**FIGURE 3:**
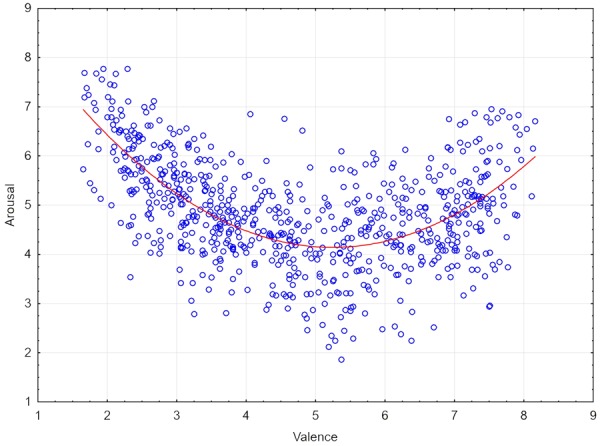
**Distribution of mean ratings of valence and arousal for 718 Polish sentences**.

To examine relationships among measured variables in more detailed fashion linear and polynomial regression analyses were performed for all the possible pairwise combinations. In **Table 4**, one can see three examples of relations that were better described by a quadratic than linear function. The first one was the relationship between valence and arousal. Inspection of the distributions of ratings in the bimodal (valence × arousal) affective space indicated that the U-shaped relationship between valence and arousal found in earlier studies was replicated in the ANPST dataset. Regression analysis with valence as the independent factor and arousal as the dependent factor was carried out to assess quadratic and linear models of the valence–arousal relationship. Results confirmed that there was a quadratic relationship between valence and arousal for assessed sentences [*y* = 0.217*x*^2^- 2.276*x* + 10.113; explained variance: *R*^2^ = 0.38, *F*(2,715) = 216.94, *p* = 0.001], whereas the linear relationship accounted for only 8% of the variance in [*R^2^* = 0.08, *F*(1,716) = 60.07, *p* = 0.001]. There was a significant change in *R*^2^ when the quadratic function was included in the model [*F*(1,715) = 344.95, *p* = 0.001]. The same analyses were repeated separately for assessments by male and female students. In both cases the valence–arousal relationship was explained better by the quadratic function [female students: *y* = 0.175*x*^2^– 1.865*x* + 9.256; *R*^2^ = 0.33, *F*(1,715) = 179.54, *p* = 0.001; male students: *y* = 0.204*x*^2^ - 2.137*x* + 9.72; *R*^2^ = 0.26, *F*(1,715) = 125.98, *p* = 0.001] than the linear function [female students: *R*^2^ = 0.09, *F*(1,716) = 73.25, *p* = 0.001; male students: *R*^2^ = 0.04, *F*(1,716) = 29.05, *p* = 0.001]. In the case of both male and female students including the quadratic function in the model produced a statistically significant change in *R*^2^ [female students: *F*(1,715) = 259.4, *p* = 0.001; male students: *F*(1,715) = 214.26, *p* = 0.001]. **Figure 3** presents the distribution of the ratings of the 718 sentences in bimodal affective space.

Two other cases when a quadratic relationship explained more variance were the dominance–arousal and the origin–valence relationships. Regression analysis with dominance as the independent factor and arousal as the dependent factor showed that the dominance–arousal relationship was quadratic for the assessed sentences [*y* = 0.163*x*^2^ – 1.683*x* + 8.895: *R*^2^ = 0.11, *F*(2,715) = 41.74, *p* = 0.001] rather than linear [*R*^2^ = 0.02, *F*(1,716) = 13.78, *p* = 0.001]; there was a change in *R*^2^ due to inclusion of the quadratic function [*F*(1,715) = 62.13, *p* = 0.001]. The same analysis was repeated separately for both sexes. In both cases, the dominance–arousal relationship was explained better by a quadratic function [female students: *y* = 0.132*x*^2^ – 1.376*x* + 8.297; *R*^2^ = 0.09, *F*(1,715) = 33.79, *p* = 0.001; male students *y* = 0.177*x*^2^ - 1.769*x* + 8.852; *R*^2^ = 0.07, *F*(1,715) = 25.99, *p* = 0.001] than a linear function [female students: *R*^2^ = 0.02, *F*(1,716) = 27.09, *p* = 0.001; male students: *R*^2^ = 0.005, *F*(1,716) = 3.55, *p* = 0.06]; in both sexes there was a change in *R*^2^ change due to inclusion of the quadratic function [female students: *F*(1,715) = 49.34, *p* = 0.001; male students: *F*(1,715) = 48.21, *p* = 0.001].

**FIGURE 4:**
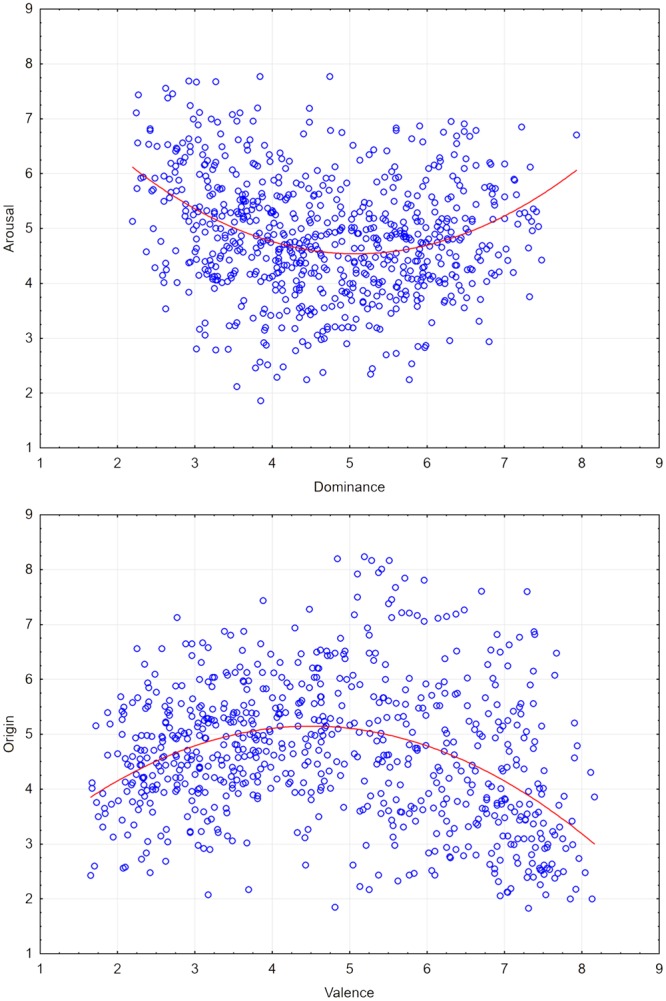
**Distribution of mean dominance and arousal ratings for 718 Polish sentences **(Top)** and mean valence and origin ratings (Bottom)**.

Regression analysis with valence as the independent factor and origin as the dependent factor showed that the valence–origin relationship was quadratic [*y* = -0.151*x*^2^ + 1.373*x* + 2.009: *R*^2^ = 0.14, *F*(2,715) = 59.6, *p* = 0.001]; the linear relationship accounted for less variance [*R*^2^ = 0.03, *F*(1,716) = 19.28, *p* = 0.001] and there was a change in *R*^2^ change due to inclusion of the quadratic function [*F*(1,715) = 99.66, *p* = 0.001]. The analyses were repeated separately for both sexes and in both sexes the valence–arousal relationship was explained better by the quadratic function [female students: *y* = –0.121*x*^2^ + 1.027*x* + 2.912; *R*^2^ = 0.12, *F*(1,715) = 46.76, *p* = 0.001; male students: *y* = –0.155*x*^2^ + 1.443*x* + 1.738; *R*^2^ = 0.12, *F*(1,715) = 46.72, *p* = 0.001] than by the linear function [female students: *R*^2^ = 0.035, *F*(1,716) = 25.63, *p* = 0.001; male students: *R*^2^ = 0.013, *F*(1,716) = 9.59, *p* = 0.002]. In both sexes there was a change in *R*^2^ due to inclusion of the quadratic function [female students: *F*(1,715) = 65.57, *p* = 0.001; male students: *F*(1,715) = 82.27, *p* = 0.001]. **Figure 4** presents the distribution of ratings of the 718 sentences in bimodal affective spaces representing dominance and arousal and origin and valence.

Considering linear correlations, the relationship between arousal and subjective significance (*r* = 0.51) is interesting and noteworthy. The positive correlation between these two dimensions may indicate that arousal and subjective significance share a common activational basis, and therefore are valid measures of two different aspects of activation. More arousing stimuli are perceived to evoke more subjectively significant experiences. Arousal was correlated negatively with origin, which confirms the hypothesis that arousal is a measure of AES activation. Origin was negatively correlated with subjective significance, which means that sentences which evoked an affective state of automatic origin also tended to carry more subjective significance that those which evoked an affective state of reflective origin. This challenges the hypothesis stating that subjective significance selectively activates the RES system. Probably the above correlation is moderated by the quadratic relationships between origin and valence and between valence and arousal. Simply put, valenced stimuli are perceived as more arousing. Similarly, valenced sentences are more automatically originated, hence one would expect stimuli which evoke automatic affective states to be more activating than those which evoke reflective affective states, as they tend to be more strongly valenced. A similar argument can be applied to the positive correlation between origin and source. Stimuli which evoked automatic affect were also perceived to evoke more internally sourced affective states and hence subjective significance was negatively correlated with source: stimuli which evoke internally sourced affective states were perceived as evoking more subjectively significant experiences than stimuli which evoked externally sourced affective states.

Considering quadratic correlations, it is worth highlighting that the U-shaped function describing the relationship between valence and arousal is commonly found in research on affective norms (e.g., Bradley and Lang, 1999a; Redondo et al., 2007; Soares et al., 2012; Monnier and Syssau, 2013; Montefinese et al., 2013, 2014; Moors et al., 2013; Imbir, 2015, 2016a; Hinojosa et al., 2016). A quadratic relationship between dominance and arousal was observed recently in the Italian version of ANEW (Montefinese et al., 2014). This relationship is easy to explain by taking into account the huge positive linear correlation between valence and dominance. In the ANPST dataset, the correlation between valence and dominance was *r* = 0.83, which means that the constructs share about 69% common variance. Very similar results were found with the Italian version of ANEW [(Montefinese et al., 2014) *R*^2^ = 0.72] and the Polish ANPW [(Imbir, 2015) *R*^2^ = 41%]. The effect suggests that positively valenced emotions are treated as controllable whereas negatively valenced emotions are perceived as rather uncontrollable; these relationships seem to be general to several languages (Bradley and Lang, 1999a; Montefinese et al., 2013, 2014; Warriner et al., 2013).

In considering the proposed new dimensions it is worth highlighting the quadratic relationship (inverted U-shape, see **Figure 4**) between origin and valence. Emotions evoked by both positively and negatively valenced sentences tended to be perceived as predominantly automatic in origin, whereas those evoked by neutrally valenced sentences were perceived as predominantly reflective in origin. This relationship is much weaker (*R*^2^ = 0.14) than in the case of valence and arousal (*R*^2^ = 0.38), but in both cases it explained more variance than the linear function. This finding may be an effect of some association between emotional (valenced) and “from the heart” aspects of the SAM scale descriptions used. The AES is more active in emotional processing than the RES, is ontogenetically earlier and is universal for all people (Jarymowicz and Imbir, 2015), whereas the RES demands more resources (Kahneman, 2011), and thus appears later in human development and is not used by all humans (Jarymowicz and Imbir, 2015). These differences between the systems imply that the AES handles many more emotional stimuli than the RES and hence the relationship between valence and origin is described better by a quadratic function than a linear function. One would not expect perceptions which appear to come “from the mind” to be as easily associated with affect as those which appear to come “from the heart.” Fortunately, the last effect was minimized by the procedure applied. Participants never assessed both valence and origin for a single sentence at once and, furthermore, these scales were separated by the arousal and dominance scale assessments.

### Comparison between Word (ANPW) and Sentence (ANPST) Datasets with Respect to Relationships between Scales

It is interesting to compare the correlation patterns obtained for sentences in the ANPST (see **Table 4**) with those observed for words constituting ANPW dataset (ANPW; Imbir, 2015). Both assessments used the same scales and a paper-and-pencil procedure, but the nature of the material assessed was different; in this study, the material included contextual information. The pattern of correlations among the classical affective dimensions (valence, arousal, and dominance) was similar in both datasets: there was a U-shaped distribution in bimodal valence × arousal affective space. The correlations obtained in the two studies were compared using Fisher’s *z*. The cocor software package (Diedenhofen and Musch, 2015) was used, as this enabled to calculate Fisher’s *z* easily, from *r-*values and numbers of observations. In both datasets (ANPW and ANPST), the highest positive correlation was between valence and dominance (ANPW: *r* = 0.64); it appears that this correlation was stronger in the ANPST dataset (*z* = 10.28, *p* = 0.0001).

The correlation between arousal and subjective significance was similar in the two datasets. In both cases the correlation was positive (ANPW: *r* = 0.24), but it was stronger in the sentence dataset (*z* = 7.06, *p* = 0.0001). Origin and arousal were negatively correlated in both datasets (ANPW: *r* = -0.2), but the correlation was stronger in the sentence dataset (*z* = -3.87, *p* = 0.0001). There were differences between the datasets with respect to correlations involving subjective significance. In the ANPW dataset this dimension was positively correlated with valence (*r* = 0.46) and dominance (*r* = 0.5), whereas in ANPST dataset subjective significance was not correlated with valence and the correlation with dominance was much weaker (*z* = -10.19, *p* = 0.0001). The opposite pattern was observed for correlations between source and valence (ANPW: *r* = 0.09; *z* = -3.78, *p* = 0.0002) and between source and dominance (ANPW: *r* = 0.1; *z* = -4.68, *p* = 0.0001). In both datasets, origin was negatively correlated with subjective significance, albeit weakly in the ANPW dataset (*r* = -0.08); the correlation was stronger in the sentence dataset (*z* = 4.85, *p* = 0.0001). Origin was also positively correlated with source in the ANPW dataset (*r* = 0.2) and this correlation was stronger in the ANPST dataset (*z* = 3.61, *p* = 0.0003). Source was similarly negatively correlated with subjective significance in both datasets (ANPW: *r* = -0.25; *z* = 1.17, *p* = 0.24). This differences may be due to the selection of materials constituting ANPST.

Interestingly, in the majority of cases the correlations between variables were stronger for sentences than for Polish words (Imbir, 2015). This may be because the contextual information provided by the sentences influenced participants’ assessments. Context may bring some additional meanings that are related to another dimensions and hence the correlations in ratings are higher. Alternatively, this may be explained by the selection of materials in the ANPST that was biased toward selecting or creating stimuli polarized on more than one dimension at once. To summarize, the similarities between the correlations observed in the two datasets suggests that both the traditional and new affective dimensions are valid.

### Comparison of Psychology Students and Non-psychology Students

Two methods were used to assess differences in the assessments of psychology students and students of other subjects. The first concerned the stability of the measurement between the two groups of participants: the psychology students (Study1a) and the students from different departments (Study 1b) assessing the subset of 108 sentences. Pearson correlations indicated that the ratings of the two groups were correlated for all dimensions (*p* < 0.001); the value of *r* varied from 0.977 for valence to 0.857 for source. Results are presented in **Table 5**.

**Table 5:** Correlations between the ratings of female and male subsamples as well as between 322 non-psychology students and psychology students (Study 1a – 1b).

Scale	Correlations with psychology students assessments (108 sentences) (Study 1a – 1b)	Female–Male subsamples correlation (Studies 1b and 2)
Valence	0.977	0.929
Arousal	0.881	0.782
Dominance	0.928	0.841
Origin	0.936	0.848
Significance	0.913	0.769
Source	0.857	0.657

The second method was an ANOVA conducted with sample (non-psychology women vs. psychology women) as a within-sentences factor and valence category [valence was chosen as it is the most intuitive dimension and was easiest to categorize, sentences were assigned to categories based on mean score: negative: 1–4; neutral: 4–6; and positive: 6–9 (Gilet et al., 2012; Monnier and Syssau, 2013)] as a between-sentences factor. Below only effects of group or interaction of valence and group will be reported, due to their relevance for sample differences. Main effects of valence will not be further discussed because the effect is quite obvious in most of the cases and irrelevant for sample differences. ANOVA with valence as the dependent variable revealed no main effect of sample [*F*(1,105) = 0.66, *p* = 0.42, η^2^ = 0.006], however, there was a main effect of valence category [*F*(2,105) = 623.35, *p* = 0.001, η^2^ = 0.92] and an interaction between group and valence category [*F*(2,105) = 10.88, *p* = 0.001, η^2^ = 0.17]. Simple main effect of sex analyses for each valence category showed that female psychology students assessed negative words (*M* = 2.65; *SD* = 0.54) more negatively than female non-psychology students did [*M* = 2.85; *SD* = 0.7; *F*(1,44) = 8.71, *p* = 0.005, η^2^ = 0.17] and positive words (*M* = 7.32; *SD* = 0.7) more positively [*M* = 6.99; *SD* = 0.56; *F*(1,46) = 18.3, *p* = 0.001, η^2^ = 0.29], whilst there was no difference in assessments of neutral words [*F*(1,15) = 0.004, *p* = 0.9, η^2^ = 0.001]. ANOVA with arousal as the dependent variable showed no main effect of sample [*F*(1,105) = 1.21, *p* = 0.27, η^2^ = 0.011], however, there was a main effect of valence category [*F*(2,105) = 29.85, *p* = 0.001, η^2^ = 0.36] and interaction between sample and valence category [*F*(2,105) = 6.44, *p* = 0.002, η^2^ = 0.11]. Simple main effect of sample analyses for each valence category showed that female psychology students assessed negative words (*M* = 6.10; *SD* = 0.94) as more arousing than female non-psychology students did [*M* = 5.6; *SD* = 0.83; *F*(1,44) = 29.89, *p* = 0.001, η^2^ = 0.41]; there were no other differences between the samples [neither for positive words: *F*(1,46) = 0.61, *p* = 0.44, η^2^ = 0.01, nor for neutral words: *F*(1,15) = 0.09, *p* = 0.77, η^2^ = 0.006]. For Dominance dimension ANOVA showed insignificant main effect of group: *F*(1,105) = 2.83, *p* = 0.096, η^2^ = 0.026, but significant main effect of valence category: *F*(2,105) = 125.7, *p* = 0.001, η^2^ = 0.71 and interaction of both: *F*(2,105) = 5.42, *p* = 0.006, η^2^ = 0.094. Simple main effect of sample analyses for each valence category showed that female psychology students assessed positive words (*M* = 6.42; *SD* = 0.85) as evoking more dominant feelings than female non-psychology students did [*M* = 5.96; *SD* = 1; *F*(1,46) = 20.97, *p* = 0.001, η^2^ = 0.31]; there were no other differences between the samples [for negative words: *F*(1,44) = 0.006, *p* = 0.94, η^2^ = 0.001 as well as for neutral words: *F*(1,15) = 0.06, *p* = 0.82, η^2^ = 0.004]. In the case of Origin dimension ANOVA showed insignificant main effect of participants group: *F*(1,105) = 0.39, *p* = 0.54, η^2^ = 0.004; but significant effect for valence category: *F*(2,105) = 27.44, *p* = 0.001, η^2^ = 0.34 and interaction of both: *F*(2,105) = 3.66, *p* = 0.03, η^2^ = 0.065. Simple main effect of sample analyses for each valence category showed that female psychology students assessed neutral words (*M* = 6.87; *SD* = 1.9) as evoking more reflective originated feelings than female non-psychology students did [*M* = 6.55; *SD* = 1.8; *F*(1,15) = 5.41, *p* = 0.03, η^2^ = 0.27]; there were no other differences between the samples [for negative words: *F*(1,44) = 0.001, *p* = 0.98, η^2^ = 0.001 as well as for positive words: *F*(1,46) = 3.45, *p* = 0.07, η^2^ = 0.07]. Subjective significance assessments ANOVA showed statistically significant main effect of group: *F*(1,105) = 17.22, *p* = 0.001, η^2^ = 0.141; valence category: *F*(2,105) = 8.74, *p* = 0.001, η^2^ = 0.14 but not significant interaction of participant’s group and valence category: *F*(2,105) = 0.56, *p* = 0.58, η^2^ = 0.01. Female psychology students in general assessed their feelings toward ANPST stimuli as more subjectively significant (*M* = 5.9; *SD* = 1.49) than female non-psychology students (*M* = 5.56; *SD* = 1.24). Last ANOVA for Source dimension assessments showed no significant main effect of participant’s group: *F*(1,105) = 0.03, *p* = 0.86, η^2^ = 0.001; but significant main effect of valence category: *F*(2,105) = 11.03, *p* = 0.001, η^2^ = 0.17 and interaction of both: *F*(2,105) = 6.02, *p* = 0.003, η^2^ = 0.1. Simple main effect of sample analyses for each valence category showed that female psychology students assessed neutral words (*M* = 6.1; *SD* = 1.78) as evoked by more external sources than female non-psychology students did [*M* = 5.55; *SD* = 1.52; *F*(1,15) = 5.05, *p* = 0.04, η^2^ = 0.25] as well as positive words (*M* = 4; *SD* = 1.27) as evoked by more internal sources than female non-psychology students did [*M* = 4.37; *SD* = 1.22; *F*(1,46) = 8.72, *p* = 0.005, η^2^ = 0.16]. There was no differences between the samples for negative words: *F*(1,44) = 0.81, *p* = 0.37, η^2^ = 0.02. **Table 6** presents mean assessments for non-psychology and psychology participants in the case of each analyzed dimension.

**Table 6:** Differences between female psychology students and female non-psychology students with respect to assessments of 108 sentences using the valence and arousal scales used in ANPST.

		Negative (*N* = 45)	Neutral (*N* = 16)	Positive (*N* = 47)	Total (*N* = 108)
		*M* (*SD*)	*M* (*SD*)	*M* (*SD*)	*M* (*SD*)
Valence	Non-psychology	2.85 (0.70)	5.17 (0.50)	6.99 (0.56)	4.99 (2.02)
	Psychology	2.65 (0.54)	5.18 (1.03)	7.32 (0.70)	5.06 (2.28)
Arousal	Non-psychology	5.60 (0.83)	3.43 (1.12)	5.14 (1.29)	5.08 (1.30)
	Psychology	6.10 (0.94)	3.35 (1.26)	5.03 (1.50)	5.23 (1.56)
Dominance	Non-psychology	3.41 (0.76)	4.69 (0.95)	5.96 (1.00)	4.71 (1.48)
	Psychology	3.40 (0.88)	4.65 (1.34)	6.42 (0.85)	4.90 (1.69)
Origin	Non-psychology	4.56 (0.91)	6.55 (1.80)	3.98 (1.36)	4.60 (1.53)
	Psychology	4.56 (0.96)	6.87 (1.90)	3.80 (1.62)	4.57 (1.75)
Significance	Non-psychology	5.47 (1.14)	4.60 (1.39)	5.98 (1.08)	5.56 (1.24)
	Psychology	5.76 (1.48)	4.84 (1.60)	6.39 (1.25)	5.90 (1.49)
Source	Non-psychology	4.84 (1.03)	5.55 (1.52)	4.37 (1.22)	4.74 (1.25)
	Psychology	4.71 (1.40)	6.10 (1.78)	4.00 (1.27)	4.60 (1.56)

The group specific differences were expected. Analysis of assessments made for ANPST stimuli by psychology students and non-psychology sample (only females) suggests that emotionally charged stimuli are assessed very similarly by both groups, especially when considering correlations and main effects of students group in comparison of mean assessments, but when considering interaction with valence some specific patterns of differences are present. This is quite an interesting finding, as most psychology experiments are conducted on first-year psychology students. The sample in Study 1a consisted mostly of second-year psychology students; they provided their ratings at the beginning of the winter term. The results were as follows: (1) there was a high correlation of assessments and (2) the only difference between the samples (main effect) was that psychology students gave higher ratings of the subjective significance of the states evoked by the stimuli. The sample difference in subjective significance ratings might be due to the introspection focus trained in the psychology population. Introspection implies meta-knowledge of the psychological mechanisms of the mind and should result in ease of subjective goals relevance perspective taking (Kahneman, 2011; Imbir, 2016b) or rational mind activation [in terms of taking additional perspective (Epstein, 2003)]. Surprisingly, results for other affective qualities such as valence, arousal, dominance, origin, and source were similar in both samples, thus contradicting the stereotype of psychology students as being especially empathetic and emotionally receptive.

The ANOVAs revealed some important interactions. Psychology students’ valence assessments were more polarized than those of other students. This may imply that psychology students are more sensitive to emotional stimulation, and therefore their reactions are slightly more pronounced in general. Also, arousal assessments for negative words were higher for the psychology major sample than for the non-psychology major sample, and dominance assessments were higher for positive words in the case of the psychology major sample. Such patterns of results suggest higher sensitivity of psychology students to negative stimuli and (probably trained) ability to treat positive experiences as more dominant (without control). Psychology students also treated neutral stimuli as more reflective in comparison to the non-psychology student sample. The neutral stimuli sample was rather small in the 108 sentence subset; therefore this result may be biased by the sentence content. They were in most cases describing natural laws, and therefore may be treated more as reflective by social science students (as associated with natural sciences), but as less reflective by other students (including natural sciences students). The last interaction of valence and sample was present in the source dimension. Psychology students perceived their reaction to neutral stimuli as more externally caused but perceived their reaction to positive stimuli as more internally caused than did non-psychology students. This also can be the result of psychology training (c.f. dominance difference for positive stimuli). The positive sentences may be more related to internal sources due to the developed ability to cherish their own life and create opportunities to feel positive emotions. All the above mentioned results allow us to state the claim that sample differences identified for the ANPST are interacting with valence of stimuli. The reason for such patterns of results may be that valence is a subjectively accessible dimension (Russell, 2003), and therefore different groups can defined by their description of what is positive and negative. Therefore, those constructs may differ in the level of other dimensions measured in ANPST. The differences may be due to initial differences between psychology and non-psychology students, but also may be result of specific training or culture norms specific for each group. Further research on this issue are needed.

The strong correlations between assessments in the two studies (using samples drawn from different populations) and the lack of direct differences (main effects) with respect to five dimensions (the exception being subjective significance) allowed us to calculate grand mean assessments for all participants. The first column of the dataset consists of weighted averages of the ratings for each of the six dimensions. Also on this basis in Study 2 some (*N* = 179) psychology students were included in the sample.

### The Effect of Sex on Emotional Evaluations

Two techniques were used to compare perceptions of affective sentences by the two sexes. The first was a Pearson correlation of ratings given by female and male students in Studies 1b and 2 (only non-psychological samples). Affective ratings were calculated separately for men and women. Ratings for all dimensions were correlated significantly (*p* < 0.001) and varied from *r* = 0.929 for valence to *r* = 0.657 for source. **Table 5** presents these correlations.

The second method was based on analyses of variance for all dimensions. Sex was treated as within-sentences factor and valence category was treated as a between-sentences factor [valence was chosen as it is the most intuitive dimension and was easiest to categorize, sentences were assigned to categories based on mean score: negative: 1–4; neutral: 4–6; and positive: 6–9 (Gilet et al., 2012; Monnier and Syssau, 2013)]. These analyses enable to investigate subtle differences in ratings and have been used in previous research to assess sex differences (Monnier and Syssau, 2013). ANOVA with valence assessments as the dependent variable revealed a main effect of sex [females assessed sentences in general as more negative (*M* = 4.67; *SD* = 1.98) than males (*M* = 4.82; *SD* = 1.58): *F*(1,715) = 13.96, *p* = 0.001, η^2^ = 0.02], a main effect of valence category [*F*(2,715) = 2966.94, *p* = 0.001, η^2^ = 0.89] and an interaction between sex and valence [*F*(2,715) = 115.73, *p* = 0.001, η^2^ = 0.25]. Simple main effect of sex analyses for each valence category showed that women tended to rate negative words (*M* = 2.7; *SD* = 0.72) more negatively than men [*M* = 3.26; *SD* = 0.64; *F*(1,294) = 259.08, *p* = 0.001, η^2^ = 0.47] and positive words (*M* = 7.11; *SD* = 0.67) more positively than men [*M* = 6.77; *SD* = 0.6; *F*(1,211) = 51.48, *p* = 0.001, η^2^ = 0.2] although there was no sex difference in valence ratings of neutral words [*F*(1,210) = 1.94, *p* = 0.17, η^2^ = 0.009]. ANOVA with arousal rating as the dependent variable revealed main effects of sex [females assessed sentences in general as more arousing (*M* = 5.05; *SD* = 1.2) than males (*M* = 4.68; *SD* = 1.03): *F*(1,715) = 155.43, *p* = 0.001, η^2^ = 0.18], valence category [*F*(2,715) = 80.29, *p* = 0.001, η^2^ = 0.18] and an interaction between sex and valence category [*F*(2,715) = 9.83, *p* = 0.001, η^2^ = 0.03]. Simple main effect of sex analyses for each valence category showed that in general women assessed words as more arousing than men did [negative words: *F*(1,294) = 129.96, *p* = 0.001, η^2^ = 0.31 (women: *M* = 5.58; *SD* = 1.16; men: *M* = 5.07; *SD* = 0.95); positive words: *F*(1,211) = 22.01, *p* = 0.001, η^2^ = 0.09 (women: *M* = 4.96; *SD* = 1.06; men: *M* = 4.72; *SD* = 0.99) as well as neutral words: *F*(1,210) = 38.31, *p* = 0.001, η^2^ = 0.15 (women: *M* = 4.38; *SD* = 1.04; men: *M* = 4.08; *SD* = 0.91)]. For Dominance dimension ANOVA showed significant main effect of sex [females assessed sentences in general as evoking less dominant experiences (*M* = 4.61; *SD* = 1.47) than males (*M* = 4.76; *SD* = 1.16): *F*(1,715) = 14.51, *p* = 0.001, η^2^ = 0.02; valence category: *F*(2,715) = 627.75, *p* = 0.001, η^2^ = 0.64 and interaction of both: *F*(2,715) = 41.34, *p* = 0.001, η^2^ = 0.1]. Simple main effect of sex analyses for each valence category showed that women tended to rate negative words (*M* = 3.41; *SD* = 0.92) as evoking less dominant experiences in comparison to men assessments [*M* = 3.84; *SD* = 0.64; *F*(1,294) = 93.34, *p* = 0.001, η^2^ = 0.24] and positive words (*M* = 6.14; *SD* = 0.94) as evoking more dominant experiences in comparison to men assessments [*M* = 5.95; *SD* = 0.73; *F*(1,211) = 12.04, *p* = 0.001, η^2^ = 0.05], although there was no sex difference in dominance ratings of neutral sentences [*F*(1,210) = 3.76, *p* = 0.054, η^2^ = 0.02].

In the case of Origin dimension ANOVA showed significant main effect of sex [females assessed sentences in general as more automatic originated (*M* = 4.59; *SD* = 1.44) than males (*M* = 4.70; *SD* = 1.16): *F*(1,715) = 15.65, *p* = 0.001, η^2^ = 0.02; valence category: *F*(2,715) = 38.66, *p* = 0.001, η^2^ = 0.1 and interaction of both: *F*(2,715) = 5.28, *p* = 0.005, η^2^ = 0.015]. Simple main effect of sex analyses for each valence category showed that women tended to rate positive words (*M* = 3.99; *SD* = 1.51) as evoking more automatic originated experiences in comparison to men assessments [*M* = 4.23; *SD* = 1.25; *F*(1,211) = 23.87, *p* = 0.001, η^2^ = 0.1], although there was no sex difference in origin ratings of negative [*F*(1,294) = 0.29, *p* = 0.59, η^2^ = 0.001] and neutral sentences [*F*(1,210) = 2.13, *p* = 0.15, η^2^ = 0.01]. Subjective significance assessments ANOVA showed statistically significant main effect of sex [females assessed sentences in general as evoking more subjectively significant experiences (*M* = 5.37; *SD* = 1.27) than males (*M* = 5.08; *SD* = 1.02): *F*(1,715) = 78.54, *p* = 0.001, η^2^ = 0.1; valence category: *F*(2,715) = 56.09, *p* = 0.001, η^2^ = 0.14 and interaction of both: *F*(2,715) = 18.54, *p* = 0.001, η^2^ = 0.05]. Simple main effect of sex analyses for each valence category showed that women tended to rate sentences as evoking more subjectively significance experiences both when they were negative (*M* = 5.67; *SD* = 1.17) in comparison to men assessments for negative sentences [*M* = 5.18; *SD* = 0.95; *F*(1,294) = 122.63, *p* = 0.001, η^2^ = 0.29] and positive (*M* = 5.67; *SD* = 1.22) in comparison to men assessments for positive sentences [*M* = 5.43; *SD* = 0.96; *F*(1,211) = 16.66, *p* = 0.07, η^2^ = 0.05]. There was no sex difference in subjective significance ratings of neutral sentences [*F*(1,210) = 1.41, *p* = 0.24, η^2^ = 0.007]. Last ANOVA for Source dimension assessments showed no significant main effect of sex: *F*(1,715) = 1.78, *p* = 0.18, η^2^ = 0.002; no significant main effect of valence category: *F*(2,715) = 2.03, *p* = 0.13, η^2^ = 0.006 nor interaction of both: *F*(2,715) = 0.54, *p* = 0.54, η^2^ = 0.002. **Table 7** presents mean assessments for female and male participants in the case of each analyzed dimension.

**Table 7:** Separate male and female means for assessments of valence and arousal for groups of negative, neutral and positive sentences.

		Negative (*N* = 295)	Neutral (*N* = 211)	Positive (*N* = 212)	Total (*N* = 718)
		*M* (*SD*)	*M* (*SD*)	*M* (*SD*)	*M* (*SD*)
Valence	Female	2.70 (0.72)	4.97 (0.75)	7.11 (0.67)	4.67 (1.98)
	Male	3.26 (0.64)	5.03 (0.58)	6.77 (0.60)	4.82 (1.58)
Arousal	Female	5.58 (1.16)	4.38 (1.04)	4.96 (1.06)	5.05 (1.20)
	Male	5.07 (0.95)	4.08 (0.91)	4.72 (0.99)	4.68 (1.03)
Dominance	Female	3.41 (0.92)	4.76 (0.95)	6.14 (0.94)	4.61 (1.47)
	Male	3.84 (0.75)	4.85 (0.79)	5.95 (0.73)	4.76 (1.16)
Origin	Female	4.68 (1.19)	5.08 (1.48)	3.99 1.51)	4.59 (1.44)
	Male	4.71 (0.91)	5.16 (1.21)	4.23 (1.25)	4.70 (1.16)
Significance	Female	5.67 (1.17)	4.64 (1.18)	5.67 (1.22)	5.37 (1.27)
	Male	5.18 (0.95)	4.58 (0.99)	5.43 (0.96)	5.08 (1.02)
Source	Female	4.68 (1.05)	4.70 (0.92)	4.52 (0.93)	4.64 (0.98)
	Male	4.70 (0.93)	4.72 (0.78)	4.60 (0.80)	4.68 (0.85)

The correlations between men’s and women’s ratings suggested that they assessed affective dimensions in a very similar way (see **Table 5**); correlations varied from 0.657 for source to 0.929 for valence. A similar pattern in correlations between men’s and women’s ratings (highest correlations for valence, then dominance and arousal) was obtained for several versions of the ANEW dataset, namely the Italian version (Montefinese et al., 2014), the Spanish version (Redondo et al., 2007) and a French version of the valence and arousal scales (Monnier and Syssau, 2013). Further ANOVAs showed sex differences in assessments of five affective dimensions (valence, arousal, dominance, origin, and subjective significance), but not for source. This is consistent with some studies (Monnier and Syssau, 2013; Montefinese et al., 2014), but others have reported no sex differences (Redondo et al., 2007). Women perceived sentences from the ANPST in general as evoking more negative, more arousing, less dominant, more automatically originated and more subjectively significant emotional reactions in comparison to men. Considering interactions of participant sex and valence, women’s ratings were more polarized than those of men (see **Table 7**) for valence and dominance; this is a common finding (Soares et al., 2012; Monnier and Syssau, 2013; Montefinese et al., 2014) and may be explained by a sex difference in sensitivity to emotionally charged stimuli or the reference point to which a stimulus is compared when valence is evaluated (Frijda, 2007; Pinheiro et al., 2016). It is possible that women are more sensitive to subtle changes in affect and thus perceive them as more intense. Women also treated stimuli in the ANPST of negative, neutral, and positive valence as more arousing and (except neutral stimuli) as more subjectively significant than men. The congruent pattern of differences for activation measures supports the claim that both arousal and subjective significance are in fact distinct factors of activation. Women were more sensitive to activation than men. Women also treated sensations as more automatically originated, but this difference was especially significant for positively valenced stimuli (c.f. **Table 7**). This may imply that women treat their sensations as more spontaneous than men. The interesting finding is lack of differences in source dimension. This may be because source assessments did not cover the whole response scale, and therefore no sufficient variability was achieved to find the actual differences to be statistically significant. Such claim is in line with the observation that the source dimension received the lowest reliability estimations; therefore one may conclude that in fact this dimension was not sufficient to assess the actual differences. Results of the current study suggest that the higher sensitivity of women to emotions is true not only for classical measures (valence, arousal, and dominance), but also new proposed dimensions.

## Limitations and Conclusion

It is worth stating that current ANPST dataset analyses have some limitations. First of all, the SAM scales allowed for measurement of subjectively perceived valence, arousal, and dominance as well as origin, subjective significance, and source. This is especially important to highlight for the newly proposed dimensions, since they are operationalizations of clearly stated dichotomies (Jarymowicz and Imbir, 2015; Imbir, 2016b). This is a methodological compromise, because given the current state of capacity to assess dual mental processes we are not able yet to measure the actual mechanism underlying the processing specific to automatic or reflective systems of evaluation. The SAM scale measures the interpretation of one’s own reaction to stimuli; therefore, the results oscillating around 5 can be interpreted as having a truly unspecified or mixed level of evaluated dimensions only when SD values are low, rather than high. In the later case, we have to deal rather with high variability of assessments. For example in the case of the source dimension, the stimulus (“Each complicated problem has a simple solution and most often it is incorrect”) corresponding to the point with a mean source value of 4.77 and a SD of approximately 1.44 (see **Figure 2**) consistently received a rating that slightly oscillated around 5 and, thus, can be defined as a stimulus that has a truly unspecified or mixed source (in other words, the entire sample of participants agreed in indicating this). On the contrary, the sentence (“There is nothing more horrible than an agonizing death”) with exactly the same mean source value, but a considerably higher SD of 3.14 likely received contrasting ratings across participants, so it would refer to a stimulus that does not have a clearly unspecified or mixed source but, rather, received mixed ratings across participants (in other words, some participants thought that it has an internal source, while some thought the opposite).

Second of all, comparisons between psychology and non-psychology students were conducted for only a subset of stimuli included in the ANPST. Additionally, the psychology student sample was chosen from an early stage of psychology education (1 year completed), thus the initial differences found in this study may be enhanced during subsequent education. The current study suggests vigilance when psychology students are invited to affective sciences experiments.

Finally, the results of the current study reveal that despite high correlations observed between assessments made by both sexes as well as psychology and other students, it is possible to find substantial differences between groups. This can be attributed to the fact that in most of the cases differences were revealed as interactions of participants’ group or sex with valence. Correlation is not sensitive to such specific differences. There were lower numbers of differences identified for field of study chosen (mainly interaction with valence of stimuli) and larger for participant sex (main effects and interaction effects were significant). This suggests that sex differences are stronger than field of study chosen as future career differences.

In conclusion, the dataset of 718 Polish short texts introduced earlier (Imbir, 2016d) and discussed here was developed to provide a lexical research tool for evoking emotions by reading emotive material that is more complex than single words (Imbir, 2015) and placed in a context. Four purely emotive dimensions—valence, dominance, origin and source—were assessed. The ANPST dataset also provides ratings of two different activation dimensions, arousal and subjective significance. The assessments were proven to be reliable and confirmed the initial expectations concerning the structure of affect in terms of classical and new proposed dimensions. The dataset properties analyses also revealed sex differences and field of study differences in emotional assessments made for sentences from the ANPST. The dataset analyzed here is a powerful research tool for all researchers interested in the impact of affectively charged stimuli on mental states and cognitive processing.

## Ethics Statement

The Maria Grzegorzewska University bioethical committee approved the design of the study and experimental conditions treatments. Participants provided their verbal informed consent to participate in this study. Written consent was not collected due to anonymity assured to participants. Any personal data of participants were not noted during the procedure. This consent procedure was suggested by the Maria Grzegorzewska University bioethical committee.

## Author Contributions

The author confirms being the sole contributor of this work and approved it for publication.

## Conflict of Interest Statement

The author declares that the research was conducted in the absence of any commercial or financial relationships that could be construed as a potential conflict of interest. The reviewer JW and handling Editor declared their shared affiliation, and the handling Editor states that the process nevertheless met the standards of a fair and objective review.

## Footnotes

1 https://figshare.com/s/e4b4e339138f07c63153
